# The association between single-nucleotide polymorphisms of TRPM7 gene and breast cancer in Han Population of Northeast China

**DOI:** 10.1007/s12032-014-0051-3

**Published:** 2014-06-21

**Authors:** Bin Shen, Lingyu Sun, Hongqun Zheng, Dongdong Yang, Jianguo Zhang, Qifan Zhang

**Affiliations:** 1 Breast Surgery of the Second Affiliated Hospital of Harbin Medical University, No. 246 Xuefu Road Nangang Strict, Harbin, 150086 Heilongjiang Province China; 2 Tumor Surgery of the Fourth Affiliated Hospital of Harbin Medical University, No. 37 Yiyuan Street Nangang Strict, Harbin, 150001 Heilongjiang Province China

**Keywords:** Breast cancer, TRPM7, Genetics

## Abstract

Breast cancer is one of the most common cancer and remains the leading cause of cancer-related deaths in women. There is increasing evidence suggesting that TRPM7 plays a pivotal role in breast cancer progression and metastasis. In this study, a case–control study was carried out to investigate the effects of SNPs in TRPM7 genes in the development of breast cancer in Han Population of Northeast China. A total of six SNPs (rs8042919, rs4775899, rs11635825, rs7173321, rs616256, and rs11070795) were chosen and genotyped. Genotypes were analyzed using a single-base primer extension assay. Chi-square (*χ* ^2^) test was used to analyze statistical difference between control and patient groups in genotype and allele frequencies. The genotype-specific risks and allele frequencies of haplotypes in breast cancer patients and controls were estimated by OR and 95 % confidence intervals. The G allele of rs8042919 was associated with a reduced disease risk. The G allele of rs7173321 and particularly its homozygous GG genotype are associated with an increased breast cancer risk. Two of the TRPM7 SNPs (rs8042919 and rs7173321) are associated with breast cancer patients in Han Population of Northeast China.

## Introduction

Calcium and magnesium ions play a central role in many cellular processes, including muscle contraction, transmitter release, cell proliferation, differentiation, gene transcription, apoptosis, and angiogenesis [1, 2]. Disordered regulation of calcium and magnesium levels may also lead to irregularities in many biological activities, even leading to carcinogenesis. Transient receptor potential melastatin 7 channels are non-selective cation channels with predominant permeability for Ca^2+^ and Mg^2+^. Activation of TRPM7 is implicated in diverse physiological and pathological processes, such as Mg^2+^ homeostasis [3–6], cell viability, growth and proliferation [7–9], anoxic neuronal cell death [10], synaptic transmission, and cell adhesion [11, 12]. It has been reported that TRPM7 is abundantly expressed in a variety of human carcinoma cells, including gastric adenocarcinoma cells [13], lung cancer cells [14], and breast cancer cells [15].

We therefore hypothesized that TRPM7 gene variation may contribute to the pathogenesis of human breast cancer. The aim of this study was to evaluate the potential association of 8 TRPM7-tagging single-nucleotide polymorphisms (SNPs) with the risk of incident in breast cancer.

## Materials and methods

### Study subjects

A total of 945 women were in the present study, consisting of 462 breast cancer cases and 483 controls that were frequency-matched on age (within 5 years) and race. All the individuals in this study were genetically independent ethnic Han Chinese from Northeast China. All the eligible cases were ascertained through histopathology and treated at the Department of Breast Surgery, Second Affiliated Hospital of Harbin Medical University between 2006 and 2010, and the control subjects were selected from the physical examination center during the same period. Inclusion criterion for the control subjects was absent of any clinical relevant malignancy at the beginning of the study. Criteria for exclusion included benign breast disease, family history of breast cancer, and the use of hormone replacement therapy. This project has been approved by the Scientific and Ethical Committee of the Second Affiliated Hospital of Harbin Medical University, and the written informed consent was obtained from all study participants. Baseline characteristics of study participants are shown in Table 1.

**Table 1:** Baseline characteristics of study participants

Variable	Cases (*n* = 462)	Controls (*n* = 483)
Age (years)	49.1 ± 7.2	47.3 ± 6.9
Cigarette smoking (*n*)	25 (5.4 %)	24 (5.0 %)
Alcohol drinking (*n*)	65 (14.1 %)	71 (14.7 %)
Age at first menstruation (years)	13.1 ± 1.2	13.4 ± 1.2
Breastfeeding (*n*)	284 (61.5 %)	311 (64.4 %)
Menopause (*n*)	145 (31.3 %)	139 (28.8 %)

### Extraction of DNA

Peripheral blood samples (5 mL) were collected from all the subjects in EDTA vacutainers and stored at −20 °C until further use. Genomic DNA was extracted using Omega blood DNA extraction kit (US) as per the instructions of the manufacturer. DNA concentrations were monitored using a Nanodrop spectrophotometer (US) and then stored at −20 °C until genotyping.

### SNP selection and genotype determination

We surveyed common SNPs from the public accessible database, the National Center for Biotechnology Information database SNP (NCBI dbSNP) supplemented by the CHB HapMap database of the International HapMap project. We selected a set of tagging SNPs that capture common variation and linkage disequilibrium (LD) structure across the TRPM7 gene using the Tagger program implemented in Haploview 4.2 software. Selection of tagging SNPs was based on a pairwise correlation coefficient (*r*-square) of 0.80 or greater—between tagging SNPs and untyped SNPs and a minor allele frequency (MAF) of 5 % or greater. A total of 6 SNPs identified [dbSNP rs8042919, rs4775899, rs11635825, rs7173321, rs616256, and rs11070795] were chosen and genotyped.

Genotypes were analyzed using a single-base primer extension assay with the SNaPshot Multiplex Kit, according to the manufacturer’s protocol (Applied Biosystems, Foster City, CA, USA). The sequences of PCR products were analyzed by electrophoresis on an ABI Prism 3730 DNA Analyzer. The primer sequences are shown in Table 2. Five percent of the selected randomly samples were re-genotyped for quality control (100 % concordance rate). Results were scored blinded as to case–control status.

**Table 2:** Description of the investigated TRPM7 SNPs

Position	SNP ID	Functional	MAF	Alleles	Primer sequences
48665962	rs8042919	Exon28	0.078	G/A	F: 5′-GCACCATTCACTGCTCATGT-3′
					R: 5′-CCATTGGTGTCCAGGTAGAA-3′
48709892	rs7173321	Intron	0.078	C/G	F: 5′-CTTGATACCATGATTCCAGATGA-3′
					R: 5′-TTTTAGGACTAAGGGATTGAAGC-3′
48640664	rs11070795	UTR-3	0.356	A/G	F: 5′-TTGCTGACTCCAGCACAGTT-3′
					R: 5′-ATATGGAGCCTAACCCTGATTC-3′
48640863	rs616256	UTR-3	0.411	G/A	F: 5′-TTGCTGACTCCAGCACAGTT-3′
					R: 5′-ATATGGAGCCTAACCCTGATTC-3′
48755015	rs4775899	Intron	0.409	C/T	F: 5′-GAGGCAGGAGAATCGACTGA-3′
					R: 5′-GGCACATTTGATTGGGTAACT-3′
48742239	rs11635825	Intron	0.078	T/C	F: 5′-ACCCAGTAGATGCCCAATAGA-3′
					R: 5′-TCCAAGGACCCTCACAGGTA-3′

### Statistical analysis

The difference in variable means (e.g., age) between control and patient groups was analyzed by Student’s *t* test. Chi-square (*χ* ^2^) test was used to analyze statistical differences between control and patient groups in genotype and allele frequencies. The genotype-specific risks and allele frequencies of haplotypes in breast cancer patients and controls were estimated as odds ratio (OR) and 95 % confidence intervals (CI). A *P* value of <0.05 was considered statistically significant. Statistical analysis was performed by SAS 9.1 for Windows (SAS Institute Inc., Cary, NC, USA).

## Results

The allele and genotype distribution of the TRPM7 SNPs are shown in Tables 3 and 4. No significant deviation from Hardy–Weinberg equilibrium was observed for any SNP. An association was observed between breast carcinoma occurrence and the presence of two SNPs, namely rs8042919 (A/G) located in exon 28 and rs7173321 (G/C) located in intron 10–11. The rs8042919 genotypic distributions were determined as 81.1 % for the GG, 18.2 % for the heterozygous GA status, and 0.7 % for the AA, respectively, in breast cancer parameters. The homozygous GG genotype was seen in lower percentage of patients with breast cancer (81.1 %) when compared to healthy women (88.2 %), and the OR for the GG versus GA+AA was [OR 0.577 (0.402–0.828); *P* = 0.003]. Frequency of G/A allele was 834/90 and 905/61 among patients and controls, respectively [OR 0.625 (0.445–0.876); *P* = 0.006]. The rs7173321 genotypic distributions were determined as 65.1 % for the CC, 28.8 % for the heterozygous CG status, and 6.1 % for the GG, respectively, in breast cancer parameters, and displayed 69.8 % for the CC, 27.1 % for the heterozygous CG status, and 3.1 % for the GG, respectively, in controls. The homozygous GG genotype was seen in high percentage of patients with case when compared to controls. Results showed that GG versus CC+CG genotypes exhibited a significant difference between patients and controls [OR 2.013 (1.061–3.820); *P* = 0.029]. Frequency of G allele was 0.205 and 0.167 between breast cancer parameters and controls, respectively [OR 0.078 (0.616–0.982); *P* = 0.034].

**Table 3:** Genotyping and allele frequency of TRPM7 gene in breast cancer cases and controls

Genotype	Cases (%) (*n* = 462)	Controls (%) (*n* = 483)	Allele	Cases (%) (*n* = 462)	Controls (%) (*n* = 483)
rs8042919					
GG	375 (81.1)	426 (88.2)	G	834 (90.3)	905 (93.7)
GA	84 (18.2)	53 (11.0)	A	90 (9.7)	61 (6.3)
AA	3 (0.7)	4 (0.8)			
rs7173321					
CC	301 (65.1)	337 (69.8)	C	735 (79.5)	805 (83.3)
CG	133 (28.8)	131 (27.1)	G	189 (20.5)	161 (16.7)
GG	28 (6.1)	15 (3.1)			
rs11070795					
AA	213 (46.1)	236 (48.9)	A	626 (67.7)	667 (69.0)
AG	200 (43.3)	195 (40.4)	G	298 (32.3)	299 (31.0)
GG	49 (10.6)	52 (10.7)			
rs616256					
GG	166 (35.9)	198 (41.0)	G	538 (58.2)	603 (62.4)
GA	206 (44.6)	207 (42.9)	A	386 (41.8)	363 (37.6)
AA	90 (19.5)	78 (16.1)			
rs4775899					
CC	141 (30.5)	148 (30.6)	C	522 (56.5)	555 (57.5)
CT	240 (52.0)	259 (53.6)	T	402 (43.5)	411 (42.5)
TT	81 (17.5)	76 (15.7)			
rs11635825					
TT	324 (70.1)	341 (70.6)	T	767 (83.0)	804 (83.2)
TC	119 (25.8)	122 (25.3)	C	157 (17.0)	162 (16.8)
CC	19 (4.1)	20 (4.1)

**Table 4:** TRPM7 gene polymorphisms and breast cancer risk

	Cases (%) (*n* = 462)	Controls (%) (*n* = 483)	OR	95 %CI	*P* value
rs8042919					
GG/GA+AA	375/87	426/57	0.577	(0.402–0.828)	0.003*
AA/GG+GA	3/459	4/479	0.783	(0.174–3.516)	0.749
G/A	834/90	905/61	0.625	(0.445–0.876)	0.006*
rs7173321					
CC/GG+CG	301/161	337/146	0.810	(0.617–1.064)	0.129
GG/CC+CG	28/434	15/468	2.013	(1.061–3.820)	0.029*
C/G	735/189	805/161	0.778	(0.616–0.982)	0.034*
rs11070795					
AA/GG+AG	213/249	236/247	0.895	(0.693–1.156)	0.396
GG/AA+AG	49/413	52/431	0.983	(0.651–1.486)	0.937
A/G	626/298	667/299	0.942	(0.776-1.143)	0.544
rs616256					
GG/AA+GA	166/296	198/285	0.807	(0.621–1.050)	0.110
AA/GG+GA	90/372	78/405	1.256	(0.899–1.755)	0.181
G/A	538/386	603/363	0.839	(0.698–1.009)	0.062
rs4775899					
CC/TT+CT	141/321	148/335	0.994	(0.754–1.311)	0.967
TT/CC+CT	81/381	76/407	1.139	(0.808–1.604)	0.458
C/T	522/402	555/411	0.962	(0.801–1.154)	0.674
rs11635825					
TT/CC+TC	324/138	341/142	0.978	(0.739–1.293)	0.874
CC/TT+TC	19/443	20/463	0.993	(0.523–1.885)	0.983
T/C	767/157	804/162	0.984	(0.774–1.252)	0.898

*OR* odds ratio, *CI* confidence interval

* *P* value <0.05 is labeled in bold

## Discussion

Breast cancer is a complex disease, which is influenced by a variety of genetic, environmental, and lifestyle factors. Genetic factor is an important contributor to breast cancer susceptibility. Recently, in unselected breast cancer patients, several genome-wide association studies (GWAS) or studies of specific candidate single-nucleotide polymorphisms (SNPs) have a number of novel genetic susceptibility variants and loci, including fibroblast growth factor receptor 2 (FGFR2), trinucleotide-repeat-containing 9 (TNRC9), mitogen-activated protein kinase kinase kinase 1 (MAP3K1), leukocyte-specific protein 1 (LSP1), mitochondrial ribosomal protein S30 (MRPS30), SLC4A7, transforming growth factor beta 1 (TGFB1), COX11, TOX3/LOC643714, estrogen receptor alpha (ESR1), Caspase-8 (CASP8), or chromosome 8q24 and 2q3, which were independently associated with an increased risk of breast cancer [16–19], and research in this area is still in continuous progress.

TRPM7 is a divalent cation-selective ion channel that is permeable to Ca^2+^ and Mg^2+^. Studies indicate that alterations in both calcium homeostasis and ion channel expression could play a key role in the regulation of processes, such as proliferation, differentiation, apoptosis, and oncogenesis. Recently, its expression was found in cancer cells, such as retinoblastoma, head and neck cells, gastric, and pancreatic cancer cells [13, 20, 21]. Guilber et al. [15] found that functional TRPM7 channel is expressed in human breast cancer cells and is related to the breast cancer cell proliferation. The study shows that TRPM7 is overexpressed in breast tumor tissues when compared to the adjacent non-tumor ones. TRPM7 is frequently expressed in the poorly differentiated and highly proliferative breast cancers (grade III, high Ki67), so it may be considered as a proliferative marker of poorly differentiated tumors [22]. TRPM7 was also found to be involved in breast cancer cell metastasis [13]. Thus, these previous reports suggest a functional involvement of the TRPM7 gene in the pathophysiology of breast cancer. To date, no study has assessed genetic variation in TRPM7 gene or its contribution to susceptibility to breast cancers. SNPs are the most common sources of human genetic variation, and they may contribute to an individual’s susceptibility to cancer.

In this case–control study, we found that the rs8042919 (A/G) and rs7173321 (G/C) polymorphisms of TRPM7 gene were associated with breast cancer risk in Han Population of Northeast China. Our results indicated the rs8042919 homozygous GG genotype was seen in lower percentage of patients with breast cancer (81.1 %) when compared to healthy women (88.2 %), and the odds ratios (OR) for the GG versus GA+AA were [OR 0.577 (0.402–0.828); *P* = 0.003]. Frequency of G allele was 0.903 and 0.937 among patients and controls, respectively [OR 0.625 (0.445–0.876); *P* = 0.006]. Therefore, we think the G allele of rs8042919 has the significant protective effect, which was associated with a reduced disease risk. A check of the SNP database reveals that the T1482I variant of the TRPM7 gene was identified in other populations (Reference SNP ID no. rs8042919) studied by Perlegen (http://www.ncbi.nih.gov/SNP) and the International HapMap Project (http://www.hapmap.org). We speculate that T1482I variant could be one of many contributory factors to cause breast cancer. In murine TRPM7, Ser-1482 is potential substrates for autophosphorylation by the C-terminus serine/threonine α-kinase domain. However, the Ile-1482 mutation found in these patients cannot be phosphorylated. On the other hand, when recombinant TRPM7 with the T1482I mutation was heterologously expressed in HEK-293 cells, channels remained functional but showed increased sensitivity to Mg^2+^ inhibition and reduced phosphorylation as compared with the wild type. A variant of TRPM7 with a missense mutation (T1482I) is found in a subset of patients with Guamanian amyotrophic lateral sclerosis (ALS-G) and parkinsonism dementia (PD-G) [23, 24]. Also, some researchers believe it is unlikely that T1482I or TRPM7 is associated with the Kii-ALS/PDC [25]. It needs to be proved whether variant of T1482I can be induced by rs8042919 polymorphism in breast cancer patients, and variant of T1482I leads to changes in the structure and function of TRPM7 gene. Also, it is our direction to work further. We hope that we can reduce the risk of breast cancer by analyzing the genetic variation of rs8042919.

Our study demonstrated that homozygous genotype GG of rs7173321 was more frequent in breast cancer patients. The G allele of polymorphism rs7173321 and particularly its homozygous GG genotype are associated with an increased breast cancer risk and thus could be risk factors for breast cancer development. Previous studies found that no significant evidence which the rs7173321 and rs8042919 polymorphisms are related to diabetes and incident ischemic stroke could be discovered [26–28]. And they hypothesize that TRPM7, as a housekeeping gene-regulating cellular magnesium metabolism, may truly have limited genetic variability [28]. However, it is surprising for us to find that there is weak association between TRPM7 genetic variants and breast cancer. We speculate that the inconsistency maybe results from small sample sizes, false positives, genetic heterogeneity among different populations, and disease.

In conclusion, the data indicated that TRPM7 genes polymorphism was likely associated with breast cancer in Han Population of Northeast China. The G allele of rs8042919 was associated with a reduced disease risk, while the G allele of rs7173321 and particularly its homozygous GG genotype were associated with an increased breast cancer risk. Thus, TRPM7 polymorphism might be one of candidates for the genetic marker to screen the risk of breast cancer.

## References

[1] Berridge MJ, Lipp P, Bootman MD (2000). The versatility and universality of calcium signalling. Nat Rev Mol Cell Biol.

[2] Wolf FI, Maier JA, Nasulewicz A (2007). Magnesium and neoplasia: from carcinogenesis to tumor growth and progression or treatment. Arch Biochem Biophys.

[3] Castillo B, Pörzgen P, Penner R, Horgen FD, Fleig A (2010). Development and optimization of a high-throughput bioassay for TRPM7 ion channel inhibitors. J Biomol Screen.

[4] Jin J, Desai BN, Navarro B, Donovan A, Andrews NC, Clapham DE (2008). Deletion of Trpm7 disrupts embryonic development and thymopoiesis without altering Mg^2+^ homeostasis. Science.

[5] Montell C (2003). Mg^2+^ homeostasis: the Mg^2+^ nificent TRPM chanzymes. Curr Biol.

[6] Schmitz C, Perraud AL, Johnson CO, Inabe K, Smith MK, Penner R, Kurosaki T, Fleig A, Scharenberg AM (2003). Regulation of vertebrate cellular Mg^2+^ homeostasis by TRPM7. Cell.

[7] Deason-Towne F, Perraud AL, Schmitz C (2011). The Mg^2+^ transporter MagT1 partially rescues cell growth and Mg^2+^ uptake in cells lacking the channel-kinase TRPM7. FEBS Lett.

[8] Inoue K, Xiong ZG (2009). Silencing TRPM7 promotes growth/proliferation and nitric oxide production of vascular endothelial cells via the ERK pathway. Cardiovasc Res.

[9] Hanano T, Hara Y, Shi J, Morita H, Umebayashi C, Mori E, Sumimoto H, Ito Y, Mori Y, Inoue R (2004). Involvement of TRPM7 in cell growth as a spontaneously activated Ca^2+^ entry pathway in human retinoblastoma cells. J Pharmacol Sci.

[10] Aarts M, Iihara K, Wei WL, Xiong ZG, Arundine M, Cerwinski W, MacDonald JF, Tymianski M (2003). A key role for TRPM7 channels in anoxic neuronal death. Cell.

[11] Visser D, Langeslag M, Kedziora KM, Klarenbeek J, Kamermans A, Horgen FD, Fleig A, van Leeuwen FN, Jalink K (2013). TRPM7 triggers Ca^2+^ sparks and invadosome formation in neuroblastoma cells. Cell Calcium.

[12] Middelbeek J, Kuipers AJ, Henneman L, Visser D, Eidhof I, van Horssen R, Wieringa B, Canisius SV, Zwart W, Wessels LF, Sweep FC, Bult P, Span PN, van Leeuwen FN, Jalink K (2012). TRPM7 is required for breast tumor cell metastasis. Cancer Res.

[13] Kim BJ, Park EJ, Lee JH, Jeon JH, Kim SJ, So I (2008). Suppression of transient receptor potential melastatin 7 channel induces cell death in gastric cancer. Cancer Sci.

[14] Gao H, Chen X, Du X, Guan B, Liu Y, Zhang H. EGF enhances the migration of cancer cells by up-regulation of TPRM7. Cell Calcium. 2011;50(6):559–68. 10.1016/j.ceca.2011.09.003 21978419

[15] Guilbert A, Gautier M, Dhennin-Duthille I, Haren N, Sevestre H, Ouadid-Ahidouch H (2009). Evidence that trpm7 is required for breast cancer cell proliferation. Am J Physiol Cell Physiol.

[16] Easton DF, Pooley KA, Dunning AM (2007). Genome-wide association study identifies novel breast cancer susceptibility loci. Nature.

[17] Ahmed S, Thomas G, Ghoussaini M (2009). Newly discovered breast cancer susceptibility loci on 3p24 and 17q23.2. Nat Genet.

[18] Stacey SN, Manolescu A, Sulem P (2008). Common variants on chromosome 5p12 confer susceptibility to estrogen receptor-positive breast cancer. Nat Genet.

[19] Dunning AM, Healey CS, Baynes C (2009). Association of ESR1 gene tagging SNPs with breast cancer risk. Hum Mol Genet.

[20] Jiang J, Li MH, Inoue K, Chu XP, Seeds J, Xiong ZG (2007). TRPM7-like current in human head and neck carcinoma cells: role in cell proliferation. Cancer Res.

[21] Yee NS, Zhou W, Lee M, Yee RK (2012). Targeted silencing of TRPM7 ion channel induces replicative senescence and produces enhanced cytotoxicity with gemcitabine in pancreatic adenocarcinoma. Cancer Lett.

[22] Dhennin-Duthille I, Gautier M, Faouzi M, Guilbert A, Brevet M, Vaudry D, Ahidouch A, Sevestre H, Ouadid-Ahidouch H (2011). High expression of transient receptor potential channels in human breast cancer epithelial cells and tissues: correlation with pathological parameters. Cell Physiol Biochem.

[23] Hermosura MC, Nayakanti H, Dorovkov MV, Calderon FR, Ryazanov AG, Haymer DS, Garruto RM (2005). A TRPM7 variant shows altered sensitivity to magnesium that may contribute to the pathogenesis of two Guamanian neurodegenerative disorders. Proc Natl Acad Sci USA.

[24] Park HS, Hong C, Kim BJ, So I (2014). The pathophysiologic roles of TRPM7 channel. Korean J Physiol Pharmacol.

[25] Hara K, Kokubo Y, Ishiura H, Fukuda Y, Miyashita A, Kuwano R, Sasaki R, Goto J, Nishizawa M, Kuzuhara S, Tsuji S (2010). TRPM7 is not associated with amyotrophic lateral sclerosis-parkinsonism dementia complex in the Kii Peninsula of Japan. Am J Med Genet Part B.

[26] Romero JR, Castonguay AJ, Barton NS, Germer S, Martin M, Zee RY (2010). Gene variation of the transient receptor potential cation channel, subfamily M, members 6 (TRPM6) and 7 (TRPM7), and type 2 diabetes mellitus: a case–control study. Transl Res.

[27] Romero JR, Ridker PM, Zee RY (2009). Gene variation of the transient receptor potential cation channel, subfamily M, member 7 (TRPM7), and risk of incident ischemic stroke: prospective, nested, case–control study. Stroke.

[28] Song Y, Hsu YH, Niu T, Manson JE, Buring JE, Liu S (2009). Common genetic variants of the ion channel transient receptor potential membrane melastatin 6 and 7 (TRPM6 and TRPM7), magnesium intake, and risk of type 2 diabetes in women. BMC Med Genet.

